# Comparison of Functional Protein Transduction Domains Using the NEMO Binding Domain Peptide 

**DOI:** 10.3390/ph3010110

**Published:** 2010-01-08

**Authors:** Khaleel Khaja, Paul Robbins

**Affiliations:** Department of Microbiology and Molecular Genetics, School of Medicine, University of Pittsburgh, Pittsburgh, PA 15261, USA

**Keywords:** protein transduction domains, NEMO-binding domain, NF-κB, delayed type hypesenDTH, arthritis

## Abstract

Protein transduction domains (PTDs), both naturally occurring and synthetic, have been extensively utilized for intracellular delivery of biologically active molecules both *in vitro* and *in vivo*. However, most comparisons of transduction efficiency have been performed using fluorescent markers. To compare efficiency of functional protein transduction, a peptide derived from IκB kinase ß (IKKß) that prevents formation of an active IKK complex was used as a biologically active cargo. This peptide, termed NEMO Binding Domain (NBD), is able to block activation of the transcriptional factor NF-κB by IKK, but not basal NF-κB activity. Our results demonstrate that Antp and Tat PTDs were most effective for delivery of NBD for inhibition of NF-κB activation compared to other PTD-NBD in both Hela and 293 cells, however, at higher concentrations (100 µM), the Antp-NBD as well as the FGF-NBD peptide caused significant cellular toxicity. In contrast to the cell culture results, delivery of NBD using 8K (octalysine) and 6R (six arginine) were the most effect in blocking inflammation following local, footpad delivery in a KLH-induced DTH murine model of inflammatory arthritis. These results demonstrate differences between PTDs for delivery of a functional cargo between cell types.

## 1. Introduction 

Protein transduction domains (PTDs) are small peptides that are able to carry larger molecules such as oligonucleotides, peptides, full-length proteins, 40-nm iron nanoparticles, bacteriophages, and even 200 nm liposomes across cellular membranes [1,2,3,4,5,6]. They have proven useful in delivering biologically active cargoes both in cell culture and *in vivo* in animal models. Interestingly, they have the ability to transduce nearly all tissues, including the brain, following intraperitoneal or intravenous administration of fusion proteins or PTD conjugates [7,8,9,10,11].

There are two general classes of PTDs described, including positively charged transduction domains (cationic) and protein leader sequence derived domains (hydrophobic), both able to transduce a wide variety of cell types. In addition, PTDs identified by phage display are able to transduce cells in a cell type or tissue specific manner that are neither cationic nor hydrophobic, presumable entering cells through a novel mechanism. We have previously characterized a panel of positively charged PTDs containing 4-12 lysines or arginines, for ability to transduce different cell types in culture [12]. We have shown that biotinylated homo-polymer peptides containing 6-8 arginines or lysines are able to deliver streptavidin fluorescent markers or ß-gal protein efficiently through a receptor independent manner [12]. In particular, 8K was the most effective for transduction of fibroblasts and epithelial cell lines in culture. However, the majority of studies evaluating the activity of PTD sequences, in particular cellular uptake, biodistribution, and endosomal escape, have used PTDs carrying fluorescent cargos. While this approach allows for analysis of tranduction efficiency and mechanism, it provides very little information about the potential for therapeutic application of this technology. Indeed, it may be possible that the majority of a specific PTD-delivery therapeutic protein is degraded prior to conferring a biological effect or never escapes the endosome. Also, the efficiency of a PTD observed in cell culture may not reflect activity *in vivo*. 

To examine *in vivo* efficacy of a variety of PTDs we evaluated their affects using a peptide sequence known to inhibit NF-κB. NF-κB is transcription factor that plays a central role in regulating the immune response as well cell survival. The aberrant regulation of NF-κB has been implicated in numerous autoimmune and inflammatory diseases including rheumatoid arthritis (RA), diabetes (DM), inflammatory bowel disease (IBD), heart disease, and multiple sclerosis (MS) [13,14,15,16,17,18,19]. Numerous cellular stress and inflammatory signals stimulate NF-κB transcription by activating the Inducible IκB Kinase (IKK) complex which is comprised of three subunits, the IKKγ (NEMO) regulatory subunit and the two catalytic subunits IKKα and β [20,21,22]. The functional interaction of these subunits allows for phosphorylation of IκB, the cytoplasmic inhibitor of NF-κB [23]. After phosphorylation, IκB is ubiquitinated and subsequently degradaded, releasing NF-κB so that it can shuttle to the nucleus and activate proinflammatory genes.

A small (11 amino acids) domain termed the NEMO binding domain (NBD) has been identified within IKKß that confers binding to IKKγ [8]. PTD delivery of NBD results in inhibition of the interaction of IKKα and β, the two catalytic subunits of NF-κB activation, with the regulatory subunit IKKγ. In particular, when this short peptide NBD (TALDWSWLQTE) was linked to Antp PTD, it led to a dose dependent inhibition of NF-κB signaling in tissue culture and in animal models. 

In the present study, we have evaluated and compared the different PTDs in a functional assay using the NF-κB inhibitor NBD peptide as a biologically active cargo. Our results demonstrate that in HeLa and 293 cells, Antp and Tat PTDs were most effective for delivery of NBD for inhibition of NF-κB activation, but the inhibition was in part due to toxicity of the Antp-NBD. Similarly, toxicity was observed with the FGF-NBD peptide. In contrast to the cell culture results, delivery of NBD using the cationic 8K (octalysine) and 6R (six arginine) peptides was the most effective in blocking inflammation following local, footpad delivery in a KLH-induced DTH murine model of inflammatory arthritis. 

## 2. Results and Discussion 

### 2.1. Transduction Efficiency and Toxicity of PTD-NBD Peptides in HeLa Cells 

**Figure 1:**
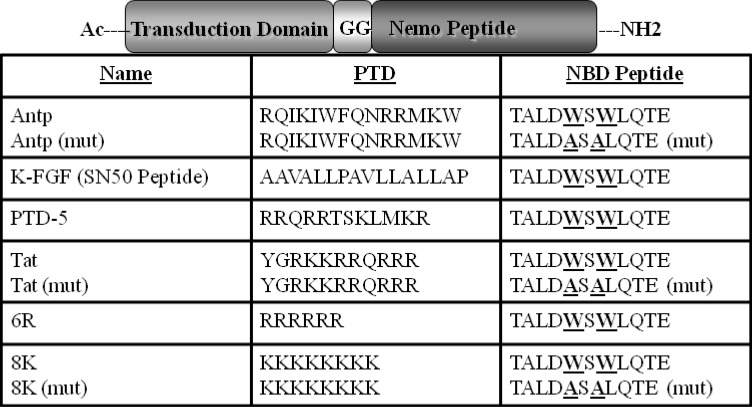
Structure of the PTD-NBD fusion peptides. The different PTDs tested as well as the sequences for wild type and mutant NBD peptides are indicated. The PTD and the NBD domain were separated by a diglycine (GG) spacer.

To assess the overall transduction efficiency mediated by the different PTDs (Figure 1) including Antp, FGF, PTD5, TAT, 6R, and 8K, flow cytometric analysis was used. Confluent HeLa cell monolayers were incubated with 6CF conjugated PTD-NBD at a concentration of 10 and 100 µM for 1 hour at 37 °C, representing the time point with maximum transduction (data not shown). 

**Figure 2:**
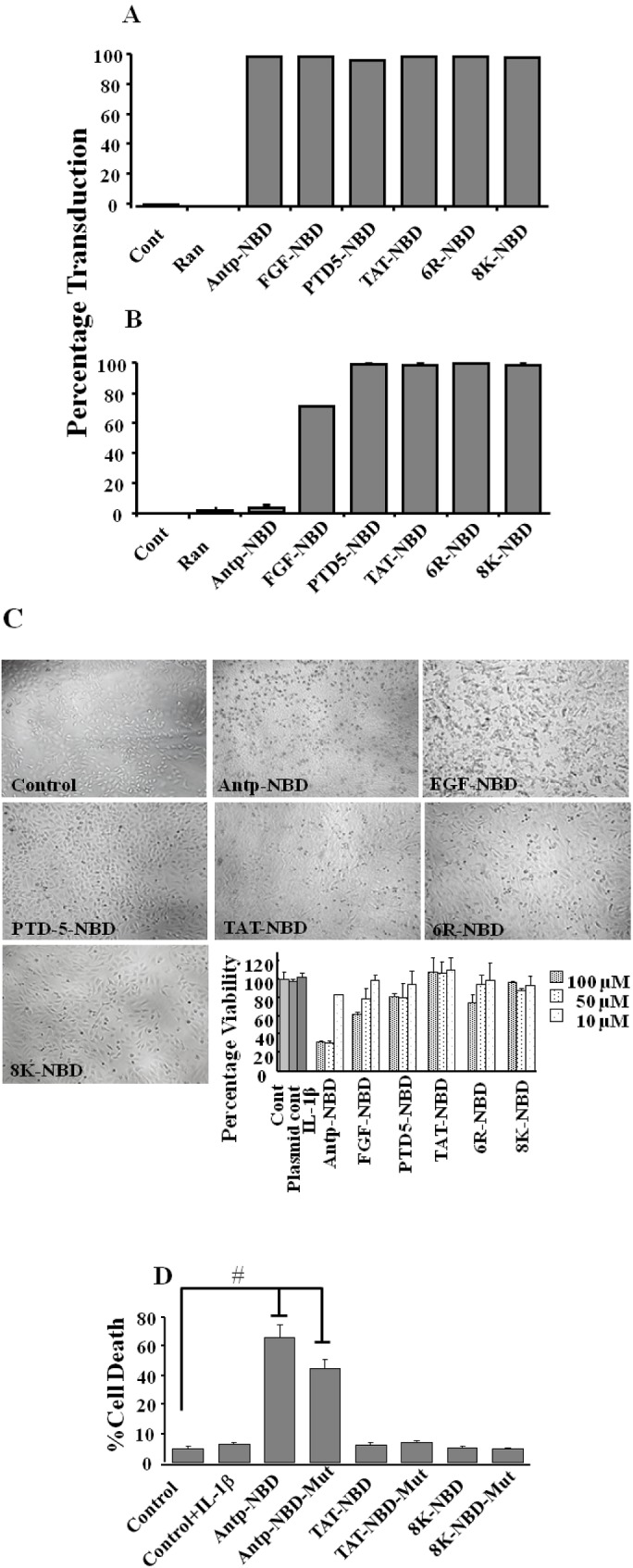
Transduction efficiency of PTD-NBD peptides. Figure showing FACs analysis of HeLa cells after 1 h of incubation with 6CF control peptide (random) or 6CF-PTD-NBD peptides. Cells were treated for 1 h with either 10 or 100 µM of fusion peptides as indicated. Cells positive for 6CF were analyzed and the % of positive cells graphed (Panels A and B). Cytoxicity of PTD-NBD peptides. HeLa cells were incubated with varying concentrations of PTD-NBD peptides, after 3 h incubation, cells were washed, evaluated by microscopy (Panel C) and viability was determined by MTT assay (panel C bar diagram; panel D). # *P* < 0.001.

Interestingly, whereas all the PTD-NBD fusion peptides transduced 100% of the cells at the 10 µM concentration (Figure 2A), there was reduced transduction in the Antp and FGF NBD peptides at 100 µM doses (Figure 2B). The morphology of the cells treated with the Antp and FGF-NBD peptides resembled that of apoptotic cell death with decreasing cellular size and nuclear fragmentation (Figure 2C). Thus toxicity of these peptides was examined using a MTT assay. Incubation of the HeLa cells with the Antp and FGF NBD peptides at concentrations of 50 and 100 µM induced significant cell death after 3 h of incubation as compared to other PTD-NBD peptides (Figure 2C bar diagram). Consistent with our transduction data, lower concentration of all peptides (10 µM) did not result in cell death. To determine if the apparent toxicity was due to NBD or to the PTD, we treated the HeLa cells with both wild type and mutant NBD PTD peptides. Treatment with both Antp-NBD wild type and mutant peptides resulted in significant cell death whereas the Tat and 8K NBD wild and mutant peptides showed no cytotoxicity (Figure 2D), even at 24 hours (data not shown). This suggests that the Antp and the FGF transduction domain peptides alone, irrespective of the cargo are toxic at high concentrations in contrast to the cationic peptides (TAT, 8K, 6R and PTD-5). A similar toxicity of the Antp peptide has been previously noted [24]. However, it is important to note that the toxicity of Antp could be, in part, cell type specific. 

### 2.2. Inhibition of IL-1ß Mediated NF-κB Activation by PTD-NBD Peptides 

**Figure 3:**
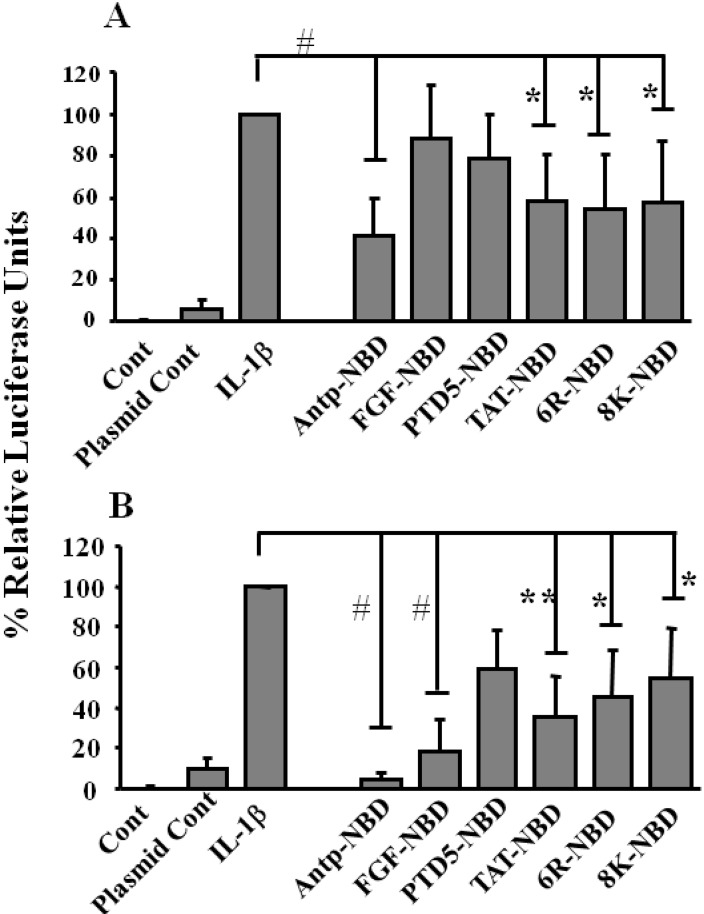
Inhibition of NF-κB activation by PTD-NBD peptides. Inhibition of the IL-1ß induced activation of the NF-κB by 10 μM (Panel A) and 100 µM (Panel B) PTD-NBD peptides. HeLa cells were transfected pNF-κB-Luc and pTK-Renilla. Cells were pretreated with 10 or 100 µM of the indicated PTD-NBD for 1 and exposed to IL-1ß for additional 3 h. The results are expressed as relative luciferase activity of three different experiments carried out in triplicate (mean ± s.d.).# *P* <0.001; ** *P*<0.01; * *P*<0.05.

To evaluate functional efficacy of the various PTDs, their ability to inhibit IL-1ß-mediated induction of the NF-*κ*B signaling pathway was examined. HeLa cells were transiently transfected with a NF-κB luciferase reporter plasmid and subsequently treated with different PTD NBD peptides prior to addition of IL-1ß. Each of the PTD-NBD peptides significantly inhibited induction of luciferase in all the groups when incubated with peptides for 3 h at 100 µM concentration (Figure 3B). In contrast, at the 10 µM concentration, the Antp, TAT, 6R and 8K-NBD peptides inhibited more effectively than the FGF and PTD5-NBD peptides (Figure 3A). However, it is likely that the inhibition observed at the higher concentration with Antp and FGF-NBD, is at least in part due to the toxicity observed at the 100 µM concentration. 

**Figure 4:**
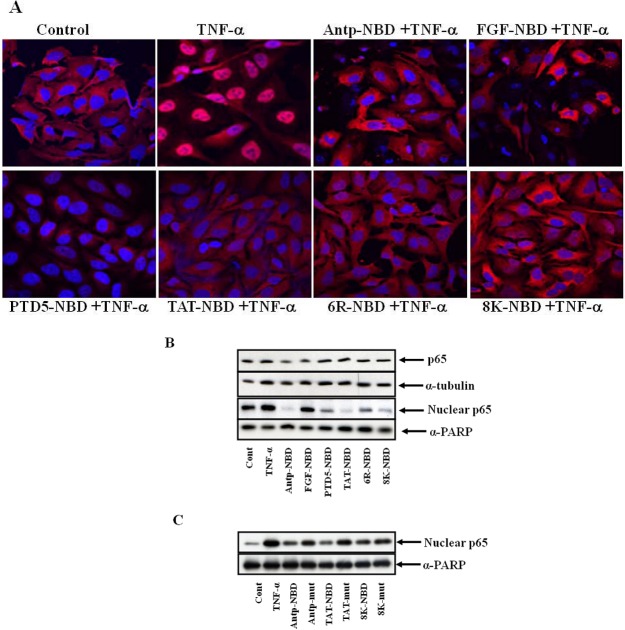
Inhibition of p65 translocation with NBD peptides. Panel A showing immunohistolochemical analysis of inhibition of p65 nuclear translocation with PTD NBD peptides**.** HeLa cells were incubated with the 100 µM of PTD-NBD peptides for 1 h, then stimulated with TNF-α (10 ng/mL) for 3 h or left untreated. The cells were fixed and immunostained for p65 (red) with nucleus counter stained (blue). (Panel B): Panel B and C showing western blot analysis of PTD-NBD peptide inhibition of TNF-α activated NF-κB binding to nucleus. 293 cells were incubated with 100 µM of PTD NBD peptides for 30 min and subsequently exposed to 10 ng/mL of TNF-α for 1h or left untreated as indicated. Cell extracts were prepared, and nuclear and cytosolic proteins separated as described in methods. The proteins were separated by SDS-PAGE and Western blotting with anti-p65 antibodies, and alpha tubulin (cytoplasmic loading control) and PARP (nuclear loading control). To confirm the NBD sequence is responsible for NF-κB suppression, 293 cells were incubated with 100 µM of PTD NBD peptides (either WT or mut attached to various PTDs) for 30 minutes and subsequently exposed to 10 ng/mL of TNF-α for 1h or left untreated as indicated (Panel C). Nuclear cell extracts were prepared, and the proteins were separated by SDS-PAGE and Western blotting with p65 and PARP, as a loading control, antibodies.

To explore further the efficacy of NBD peptides, the inhibition of nuclear p65 translocation in HeLa and 293 cells was analyzed via immunohistochemistry and immunoblotting. Initially, Hela cells were incubated with PTD NBD peptides for 1 hour, subsequently challenged with TNF-α for 3 h and p65 translocation to nucleus was determined by IHC (Figure 4A). In general, the analysis of translocation demonstrated that all the PTD-NBD peptides completely inhibited p65 translocation to nucleus. 

The level of p65 in cytosolic and nuclear fractions from the treated HeLa cells was examined by Western analysis. There was no significant difference in the level of cytosolic p65 after treatment with different PTDs NBDs. However, Antp, Tat and 8K-NBD were the most effective in blocking nuclear translocation of p65 (Figure 4B). To confirm that the inhibition of nuclear p65 translocation was due to NBD peptide and not the PTD sequence, PTDs linked with both NBD wild type (wt) and mutant NBD (mut) peptide (an inactive peptide with a mutation of two amino acids) were evaluated. As shown in Figure 4C wild type NBD peptides significantly inhibited nuclear p65 translocation as compared to control mutant peptides. 

### 2.3. Evaluation of Efficacy of the Different PTD-NBD Fusion Peptide on Inflammation in a Footpad Model of DTH 

To compare the efficacy of the different PTDs for functional delivery of NBD *in vivo*, we evaluated the PTD-NBD peptides functionally in a foot pad model of delayed-type hypersensitivity (DTH). In this model, mice are immunized to a specific antigen (KLH) and then inflammation is induced by local injection of the antigen into the foot pad, resulting in an increase in footpad size. 

**Figure 5:**
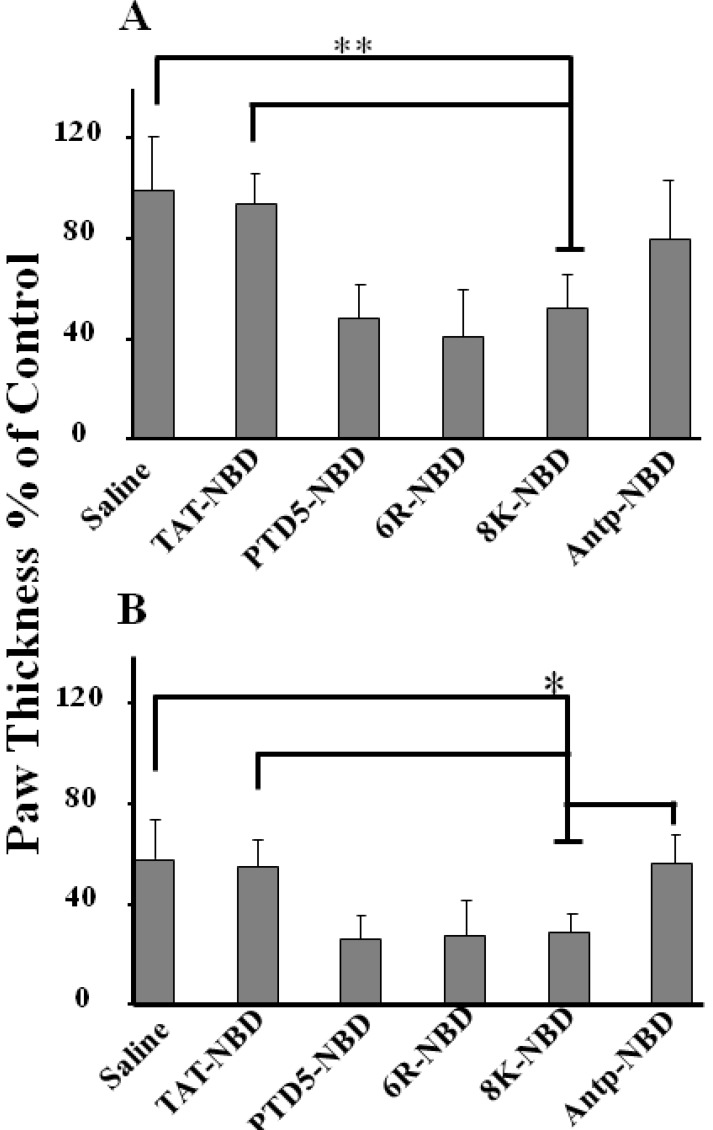
Local administration and in vivo efficiency of PTD-NBD peptides in reduction of paw swelling in treated paws of DTH mouse. Analysis of therapeutic efficacy of NBD peptides in murine DTH arthritis model. Fifty µL of 200 µM PTD-NBD peptides (25 µg/paw) was injected in to right paw 2 h before antigen administration. Mice were monitored for paw thickness at 48 (a) and 72 h (b) after antigen administration (*n* = 10). Results were expressed as the percentage of control paw swelling. ** *P* < 0.001; * *P* <0.05.

Earlier studies in mouse model of collagen induced arthritis (CIA) injection of NBD peptide significantly ameliorated the arthritis [25]. In our studies injection of the different PTD-NBD peptides was found to inhibit edema formation at 48 (Figure 5A) and 72 h (Figure 5B) after KLH injection whereas the saline control had no discernible effect. Interestingly, eight lysine (8K) and six arginine (6R) two synthetically derived sequences as well as PTD-5, worked far more efficiently in blocking footpad swelling following local injection in contrast to Tat–NBD and Antp–NBD (Figure 5A and Figure 5B).

### 2.4. Discussion

Previous studies have used predominantly fluorescently tagged PTDs for evaluation of transduction efficiency. However, the methods used for analysis of transduction such as confocal or flow cytometry, especially in fixed cells, doesn’t provide insight into the biological efficacy of the PTD. Thus we have used a biologically active therapeutic peptide, the NEMO binding domain (NBD) peptide, for inhibition of inducible NF-κB activity as an assay for functional protein transduction. In this study, we tested a panel of PTD–NBD fusion peptides for their overall transduction activity, toxicity and ability to inhibit induced NF-*κ*B transcriptional activity. The panel of PTDs tested included both cationic peptides as well as the Antp and FGF transduction domains. 

In culture, delivery of NBD with Tat, Antp and PTD-5 in HeLa cells resulted in the most effective inhibition of IL-1β induced NF-κB activity. However, Antp and FGF-NBD resulted in cell toxicity while the cationic PTD-NBD peptides showed only negligible cytotoxicity. The toxicity mediated by Antp and FGF appeared to be mediated through an apoptotic pathway, at least in part, since we observe PARP cleavage at later time points and the cells were positive for Annexin V staining. In contrast, the cationic peptides did not confer any observable toxicity in cell culture with Tat-NBD and 8K-NBD conferring the most inhibition of NF-κB in regard to inhibition of NF-κB promoter activity and p65 translocation. However, the cell culture results are in contrast with *in vivo* analysis in a murine footpad DTH model where the 8K-NBD and 6R-NBD peptides, worked far more efficiently in blocking footpad swelling following local injection. In the DTH model, injection of Tat-NBD and Antp-NBD was significantly less effective in inhibiting footpad swelling. Although the reasons for the difference in activity observed in cell culture and *in vivo* are unclear, it most likely reflects differences in the cell types transduced or the disease model. It is important to note that we have already demonstrated differences in transduction efficiency between cell types, using a fluorescent-based assay, depending in part of the levels of glycosaminoglycans on the cell surface. Even though the exact targets for the 8K and 6R-NBD peptides *in vivo* for conferring the significant therapeutic effects are poorly defined, we speculate that the 8K-NBD peptide is efficient *in vivo* at transducing and inhibiting IKK in macrophages and DCs, resulting in suppression of inflammation [26,27]. However, at least in culture, the TAT PTD is able to transduce macrophages and dendritic cells efficiently (data not shown). Taken together, our results clearly demonstrate differences in efficacy between PTDs *in vivo* and that transduction efficiency in culture do not necessarily reflect efficacy *in vivo*. 

Based on the results presented here demonstrating effective treatment of a footpad DTH model with 8K-NBD, we used the 8K-NBD peptide in the IL-10^-/-^ mouse model of inflammatory bowel disease [28]. In this model, intraperitoneal administration of 8K-NBD resulted in almost complete suppression of disease pathology in the gut of the IL-10^-/-^ mice [28] and reducing TNF-α and IL-12 expression. Moreover, our preliminary experiments suggest 8K-NBD is more effective than Tat-NBD in treating pathology in murine IBD. Similarly, it has been reported that the Antp NBD peptide is therapeutic in a murine *mdx* model of Duchenne muscular dystrophy, improving muscle pathology [29]. Our results also suggest that 8K-NBD is more therapeutic than Antp-PTD in blocking muscle degeneration and improving regeneration in mdx, in particular in diaphragm muscle. Finally, we have observed therapeutic effects of systemic administration of 8K-NBD in mouse models of accelerated aging, collagen induced arthritis and autoimmune diabetes. Taken together, these results confirm the strong *in vivo* therapeutic activity of the 8K PTD for delivery of NBD. Moreover, for at least certain disease, 8K-NBD appears to be more effective than Antp and Tat-NBD. 

## 3. Experimental Section

### 3.1. Animals

BALB/c mice were purchased from Jackson Laboratories (Bar Harbor, ME, USA). All animals were housed at the University of Pittsburgh, Center for Biotechnology animal facility in compliance with the United States Department of Agriculture and National Institutes of Health regulations. All animal manipulations were conducted and monitored under protocols reviewed and approved by the Institution Animal Care and Use Committee. 

### 3.2. Peptide Synthesis

NEMO binding domain (NBD) peptides conjugated with the different PTDs (Figure 1) were synthesized by the peptide synthesis facility (University of Pittsburgh) by the solid-phase procedure on an automated peptide synthesizer (PerSeptive Biosystems, Inc., Framingham, MA, USA) using *N*-(9-fluorenyl)methoxycarbonyl (Fmoc) synthesis protocols. Peptides were subsequently purified and characterized by reversed-phase high performance liquid chromatography and mass spectrometry. The PTD and the NBD domain were also separated by a diglycine (GG) spacer. The sequences of the wild-type and mutant (MUT) NBD peptides have been described previously [8]. The NBD peptide contains the region of IKK-β from T735 to E745 synthesized in tandem with a membrane permeabilization sequence (Figure 1) and the MUT peptide is identical except that W739 and W741 are replaced by alanines to render it biologically inactive. 

### 3.3. Flow Cytometric (FACS) Analysis of Transduction Efficiency of 6-Carboxy Fluorescien (6CF) PTD-NBD Peptides

HeLa cells were plated onto 12 well plates and cultured in Dulbecco’s modified Eagle’s media with 10% FBS, 2 mM glutamine, and 1% penicillin/streptomycin solution. The next day the cells were incubated with 10 and 100 µM of each peptide individually in duplicate for 1 hr at 37 °C, subsequently cells were washed thoroughly with PBS and were dissociated with enzyme free Hanks cell dissociation buffer (Invitrogen). The cell pellets were washed thoroughly again three times in PBS, subsequently, the transduction efficiency of panel of 6CF-PTD-NBD peptides (Figure 1) in HeLa cells were analyzed by FACS.

### 3.4. Analysis of Cytotoxicity of PTD-NBD Peptides Using MTT Assay Protocol 

The HeLa cells were grown in a 96 well plate and incubated with panel of PTD-NBD peptides for 3 h and subsequently the cells were washed and viability was determined by incubating with MTT [3-(4,5-dimethylthiazol-2-yl)-2,5 diphenyltetrazolium bromide]. The number of surviving cells is directly proportional to the level of the formazan product created. The color was read on a multi well scanning ELISA reader. 

### 3.5. Transient Transfection Assay Using NF-κB Luciferase Reporter 

The NF-κB *cis*-reporter luciferase plasmid was purchased from Stratagene (La Jolla, CA, USA). The HeLa cells were plated in to a 6 well plate in 2 ml of culture medium (DMEM, 10% FBS, 1% penicillin/streptomycin, and 1% L-glutamine). The following day, cells were transfected with a cocktail of DNA consisting of luciferase reporter DNA and internal control pTK-Renilla (Promega) using Lipofectamine 2000 following the manufacturer’s instructions. After overnight incubation, fresh medium (Opti-Mem) was added to the plates, and cells were incubated with peptides and 60 min later, 5 ng/mL IL-1ß (R&D Systems). The cells were incubated for a further 3 h, after which cells were harvested and luciferase values were measured using the dual luciferase assay kit (Promega). Each individual assay was performed in triplicate per experiment and normalized against the Renilla control.

### 3.6. Immunofluorescence Staining of p65 and Confocal Microscopy 

Hela Cells were seeded cover slips in a six well plate and cultured as described earlier. The cells were incubated with PTD NBD peptides or controls for 1 h and subsequently treated with TNF-α for 3 h. The cells were washed, and fixed in cold 2% PFA for 10 min at RT. The slides were then washed extensively with PBS and incubated with optimal dilutions of the primary antibody in TBS containing 1% FCS and 1% human serum for 60 min at room temperature, followed by 45 min incubation at room temperature with Cy3-conjugated goat-anti-rabbit IgG and Draq5 (Molecular Probes) and for each fluorochrome label, negative control antibodies were included. Finally, the slides were mounted in Vectashield (Vector Labs, Burlingame, CA, USA) for confocal microscopy analysis and p65 were visualized using a Leica (Leica Microsystems, Heidelberg, Germany) confocal system, equipped with an Ar/Kr/HeNe laser combination. Images were taken using a X40 1.25 NA objective.

### 3.7. Peptide Treatment and NF-κB Nuclear Translocation Assay 

HeLa cells were maintained in Dulbecco’s modified Eagle’s media with 10% FBS, 2 mM glutamine, and 1% penicillin/streptomycin solution. The cells were incubated with PTD-NBD peptides or controls for 30 min and subsequently treated with TNF-α for 1 h. The cells were washed and the effect of PTD-NBD peptides on the nuclear translocation of p65, in response to TNF-α, was examined by immunoblotting.

### 3.8. Preparation of Cytosolic and Nuclear Extracts 

All of the extraction procedures were performed on ice with ice-cold reagents. The treated cells were scrapped in cold PBS and were centrifuged at 2,000 *g* for 2 minutes at 4 °C and pellets were re-suspended with lysis buffer containing an anti-protease cocktail and the cytosolic and nuclear extracts were prepared using NEPR kit was obtained from Pierce (Rockford, IL, USA). The cytosolic and nuclear pellet was flash frozen in liquid Nitrogen and the samples were stored at -80 °C. Protein concentration was determined using a protein assay kit (Bio-Rad, Hercules, CA, USA).

### 3.9. Immunoblotting 

Crude cell lysates were boiled in the presence of 4X SDS-sample buffer (0.5 M Tris-HCl (pH 6.8), 10% (w/v) SDS, 10% glycerol, 0.05% (w/v) bromophenol blue, and distilled water) for 5 min and subjected to electrophoresis on 12% SDS-PAGE. Proteins were transferred to Immobilon-P membranes (Millipore, Bedford, MA, USA) according to the manufacturer’s instructions using a semi-dry blotter (Bio-Rad, Richmond, CA, USA). Subsequently, the membranes were incubated in blocking solution for 2 h (10% skim milk prepared in PBS containing 0.05% Tween 20), to reduce nonspecific binding. Membranes were washed with a PBS/Tween buffer and exposed to primary Abs overnight at 4 °C (1:1,000 dilution), washed again four times, and incubated with the respective secondary HRP-conjugated Abs for 1 h at room temperature (1:5,000 dilution). Membranes were washed extensively 3-4 times (10-15 min), and an ECL detection assay was performed following manufacturer’s directions. 

### 3.10 Induction of Delayed-Type Hypersensitivity (DTH) and PTD-NBD Treatment 

Animal experiments were approved by the Institutional Animal Care and Use Committee of University of Pittsburgh. C57BL/6 mice were sensitized on day 0 by injecting 100 ug antigen (KLH) emulsified 1:1 in Freund's complete adjuvant (FCA), intra-dermally at the base of the tail. Two weeks later, presensitized mice were given injections in one rear footpad with either 50 µL of PTD-NBD in saline or vehicle. Two hours later, the mice were challenged in both rear footpads by injecting 20 µg of antigen (KLH) dissolved in 50 µL of PBS [30]. 

### 3.11. Measurement of DTH 

The thickness of each hind paw was measured with a spring-loaded caliper (Dyer) 48 and 72 h [30]. Results were expressed in percentage of control paw swelling before and after antigen boost injection by an investigator who was blinded to the treatment groups.

### 3.12. Statistical Analysis 

Statistics were performed using the Stata 8.2 (STATA Corp., College Station, Texas) software package and data collected were expressed as mean ° SEM. A *p* value of less than 0.05 by ANOVA and long rank test analysis was used to indicate statistically significant differences.

## 4. Conclusions

Here we have compared efficiency of different protein transduction domains (PTDs) for functional delivery of peptide (NBD), able to block activation of the transcriptional factor NF-κB by IKK, but not basal NF-κB activity. We demonstrate that Antp and Tat PTDs were most effective for delivery of NBD for inhibition of NF-κB activation compared to other PTD-NBD in both HeLa and 293 cells.  However, we also demonstrate that at higher concentrations, the Antp and FGF PTDs caused significant cellular toxicity. In contrast to the cell culture results, the 8K and 6R PTDs were the most effective in blocking footpad inflammation in a DTH murine model of inflammatory arthritis. Taken together, these results demonstrate differences between PTDs for delivery of a functional cargo between cell types. The results also demonstrate the efficacy of the 8K PTD for functional delivery of NBD *in vivo*, a result that has been confirmed using 8K-NBD in mouse models of muscular dystrophy (*mdx*), accelerated aging (ERCC1-deficient), inflammatory bowel disease (IL-10^-/-^), type 1 diabetes (NOD) and collagen-induced arthritis (CIA) in Dba1 mice. 
